# Supplementary material for: Multiple associative structures created by reinforcement and incidental statistical learning mechanisms
Source: Nat Commun. 2019 Oct 23;10:4835. doi: 10.1038/s41467-019-12557-z (PMC6811627; doi:10.1038/s41467-019-12557-z)
Supplement: Supplementary file 1 — Supplementary Information [file 41467_2019_12557_MOESM1_ESM.pdf]

Supplementary Information

Multiple associative structures created by reinforcement and incidental statistical learning mechanisms

Klein-Flugge et al.

Supplementary Methods

Binary choices completed following the behavioral learning task were examined to probe stimulus preferences. Choices were grouped into two categories: those between any rewarded sequence and control sequence elements (RewSeq vs ConSeq, i.e., any of A, B, C or D versus any of A', B', C', or D'); and those between elements of the same sequence (RewSeq vs RewSeq or ConSeq vs ConSeq, e.g., A vs C or B' vs D'). The first analysis looked at the percentage of times A, B, C, or D had been chosen on day 1 in RewSeq vs ConSeq choices, averaged across contexts (**Suppl Fig 1d**, left; performance was at ceiling on day 2). The second analysis examined RewSeq vs ConSeq choices across learning blocks on day 1 and 2 separately for the three contexts, but averaged across sequence elements A, B, C, and D (**Suppl Fig 1d**, right). The third analysis focused on RewSeq vs RewSeq and ConSeq vs ConSeq choices for the four sequence elements A, B, C, and D (averaged across learning blocks and contexts, **Suppl Fig 1e**).

Finally, the percentage of times the correct sequence was produced during the completion phase was calculated based on the average across contexts and repetitions, resulting in a 1x8 ANOVA with factor block (**Suppl Fig 1g**).

Supplementary Note 1: Choices reflect sequence knowledge

After each learning block, participants were given choices between combinations of two highlighted stimuli on the 3x4 map to probe their preference for elements of the rewarded sequence. This was a way of implicitly assessing their liking of different stimuli, which was thought to reveal subtle preferences that might occur in the absence of full sequence knowledge. Crucially, no feedback on performance was provided at any point during training.

Participants average performance on choices between elements of different sequences, specifically one element of RewSeq and one of the other eight stimuli on the 3x4 map was above 90% for all elements (A, B, C, or D vs one of the other eight elements) and therefore close to ceiling. A 2x3x4 repeated-measures ANOVA with factors day, context, and block revealed an effect of day with better performance on day 2 ($F(1,25)=13.666$, $p=0.001$), an effect of block showing an improvement across blocks ($F(3,75)=14.954$, $p<0.001$), and an interaction between day and block with stronger improvements across blocks on day 1 compared to day 2 ($F(3,75)=16.64$, $p<0.001$; winning model in Bayesian repeated-measures ANOVA has effects of Day, Block and Day*Block: $P(M|data) = 0.863$, $BF_m=113.714$, $BF_{10}=1$; **Suppl Fig 1d**, right). This showed that participants had acquired robust knowledge of which elements belonged to the rewarded sequence.

Supplementary Note 2: Choices reveal preference for later sequence elements and suggest backwards learning

We probed choices between two elements of the same sequence to see if within the same sequence, there was a preference e.g. for the starting elements or elements closer to reward. Comparing the percentage of choices for each of A ,B, C, D for RewSeq versus ConSeq revealed no main effects but a significant interaction of sequence type x sequence element (4x2 repeated-measures ANOVA with factors sequence element A, B, C, D and sequence type RewSeq vs ConSeq; $F(3,75)=7.26$; $p<0.001$; Bayesian repeated-measures ANOVA in favor of full model: $p(M|data)=0.959$, BFm=94.294; **Suppl Fig 1e**). Further inspection revealed a preference for elements C and D over A and B for the rewarded sequence, and thus elements that occurred closer in time to reward. This effect was particularly clear after only one training session and thus earlier in learning. By comparison, for the ConSeq, there was a weak preference for earlier elements (A and B over C and D; **Suppl Fig 1e**). This provided behavioral evidence to suggest that the presence of reward recruited a different learning mechanism, whereby elements closest to reward get assigned the credit initially. By contrast, statistical knowledge – as for the ConSeq – was likely constructed in a forward manner.

Supplementary Note 3: Behavioral evidence for statistical learning

We probed whether participants showed evidence for incidental learning which should be unrelated to reward and driven by repeated observation of statistical relationships, such as the transitions between control sequence stimuli. We returned to the RTs for the first movement that initiates transitioning between two stimuli of the control sequence during the pre-scan learning task (grey bars in **Fig 1c**). We asked whether initiating the button press from A' to B', followed by B' to C' and C' to D' would show RT speeding because transitions, despite never being associated with reward, became increasingly predictable. Note that these transitions were experienced as frequently as those in the rewarded sequence. Progressive RT speeding, in the absence of reward during pure statistical learning, has previously been reported. For example when learning triplets XYZ, the RT to Y after X is slightly faster and the RT to Z after XY is fastest[1–4]. Consistently, a 1x3 repeated-measures ANOVA with factor Transition (A'B'/B'C'/C'D') run only on the control sequence transitions showed a significant effect of transition ($F(2,50)=28.553$, $p<0.001$; Bayesian repeated-measures ANOVA shows evidence for a model with factor Transition: BFm=1.522e+6, $P(M|data)=1$). Thus RTs became faster as participants progressed through the control sequence, but as shown in the main text, this speeding was less pronounced than in the rewarded sequence.

Supplementary Note 4: Consolidation of sequence knowledge between session 1 and 2 of the pre-scan learning task

We tested whether participants consolidated their sequence knowledge between the first and the second session of the pre-scan learning task, prior to scanning. If participants were able to anticipate the next sequence element better during the second learning session, they should be faster to respond to the first movement that initiates the path from A to B, B to C, or C to D. We therefore repeated the original ANOVA with an additional factor of day (2x2x3 repeated measures ANOVA with factor day (day1/day2) sequence (RewSeq/ConSeq) and transition (AB,BC,CD)). In addition to the effects of transition, sequence and their interaction already reported in the main text, this analysis revealed a main effect of day ($F(1,25)=55.44$, $p<0.001$), with overall faster RTs on day 2; there was also an interaction between day and sequence type ($F(1,25)=11.15$, $p=0.003$; **Suppl Fig 1f**) which was due to a larger RT improvement on day 2 for RewSeq compared to ConSeq elements. Although the triple interaction of day x sequence x transition reached significance in a conventional ANOVA ($F(2,50)=8.304$, $p<0.001$), this interaction was not included in the winning model obtained from a Bayesian repeated-measures ANOVA (Model with Day + Sequence + Transition + Day*Sequence + Transition*Sequence $P(M|Data) = 0.587$, $BF_m=25.59$, $BF_{10}=1$). Overall, this suggests that both explicit knowledge of the RewSeq as well as implicit knowledge of the unrewarded ConSeq was consolidated from the first to the second session of the pre-scan learning task, with boosted consolidation due to reward.

Supplementary Note 5: Post-scan memory of stimuli reveals differences between RewSeq and ConSeq specific to the recognition of correct stimuli

We tested participants memory for the 3x4 layout in a short memory block performed at the very end of fMRI testing (i.e., after both scans had been completed). One stimulus was probed at a time and the others greyed out, and it was shown in its correct location and color (recognition of correct stimuli), or in a wrong location or wrong color (error detection of wrong stimuli). Participants' percentage correct scores for judging these stimuli as 'correct' versus 'wrong' was entered into a 2x2 repeated-measures ANOVA with factors sequence (RewSeq/ConSeq) and type of recall (recognition/error detection). This analysis showed effects of sequence ($F(1,25)=30.20$, $p<0.001$), with better memory for RewSeq over ConSeq, and an interaction of sequence x type of recall ($F(1,25)=20.16$, $p<0.001$)), showing differences in memory between RewSeq and ConSeq were more pronounced when stimuli were shown in their correct position/color (**Suppl Fig 4e**). While type of recall only showed a trend ($F(1,25)=3.49$, $p=0.074$), the winning model in a Bayesian repeated measures ANOVA was the full model, including both main effects and interaction ($P(M|data)=0.979$,

BFm=185.80), suggesting that reward boosted memory formation and that an error could be detected more easily than a correct stimulus recalled.

Supplementary Note 6: rr-cc is capturing cross-stimulus suppression effects

The (rr-cc) contrast was designed to capture associations between rewarded sequence elements via cross-stimulus suppression. However, it may also have be driven by a main difference in BOLD activity between rewarded and control sequence elements. To confirm our interpretation as cross-stimulus suppression effects, we first examined the impact of the temporal delay between successive sequence elements. Neural adaptation effects are expected to scale with the temporal delay between stimuli, with stronger suppression and thus a smaller BOLD signal for stimuli presented closer in time. We therefore modelled the temporal delay between occurrences of rewarded sequence elements and the temporal delay between occurrences of control sequence elements parametrically. We extracted parameter estimates from regions of interest (ROIs) defined as spheres around peak coordinates in the above contrast (see Methods and **Supplementary Figure 2c**). Even though this is a very demanding test that is rarely performed in repetition suppression experiments we found that BOLD activity for the second of two successive elements of RewSeq (rr) but not ConSeq (cc) indeed scaled with the temporal distance between the stimuli in two of our four ROIs: temporal pole and amyg/hippo (temporal distance rr: $p(mPFC)=0.83$; $p(pOFC)=0.79$; $p(tempPole)=0.049$, $t(25)=1.72$; $p(amyg/hippo)=0.008$, $t(25)=2.54$; temporal distance cc: all $p>0.1$; one-sample t-tests; **Figs 2c,d and Supplementary Figure 2a**). This bolstered our interpretation of a shared neural representation of rewarded sequence elements in these regions. In a second test, we confirmed that none of our ROIs were simply showing a main effect difference between rewarded and control elements. We examined rewarded and control elements when they were preceded by an element not part of their own sequence (xr or xc). None of our ROIs showed a significant difference for the contrast xr-xc (one-sample t-tests: all $p>0.1$; **Supplementary Figure 2b**). Thus, our ROI-defining contrast captured relationships between pairs of stimuli as probed by cross-stimulus suppression, rather than differences in the overall BOLD main activation to rewarded and control stimuli.

Supplementary Note 7: Sequence fusion effects are not driven by correctly ordered (forward) pairs

Our ROI-defining sequence fusion contrast (**Fig 2**) examined repeated presentations of stimuli from the rewarded sequence (rr) with repeated presentations of any two control stimuli (cc). The rationale was that if stimuli from the rewarded sequence have more overlapping neural representations, this should lead to more cross-stimulus suppression, compared to control sequence stimulus repetitions (contrast: rr-cc). To ensure that the observed effects were not driven by only the correctly ordered

forwards pairs, we repeated the analysis but split rr and cc pairs into repetitions that were forwards-directed (ForwPair: AB, xBC, xxCD) and other within-sequence pairs that were not forwards-directed in the correct order but still transitions within the same sequence (OtherWithinPair: BA, CB, DC, AD, DA, AC, CA, BD, DB). **Suppl Fig 2e** shows the effects separately for these two sub-groups of trials. Note that forwards pairs exclude occurrences of ABC and ABCD, i.e., those where full third- or fourth-order sequence relationships were fulfilled. Suppression for rewarded over control pairs was present for both ForwPairs and OtherWithinPairs. If anything, suppression was stronger for the OtherWithinPairs. While testing each effect individually (rr-cc for just ForwPairs or rr-cc for just OtherWithinPairs) is not orthogonal to our ROI selection, the interaction between the effect of rr-cc in ForwPairs compared to OtherWithinPairs is orthogonal to ROI selection. This is true in general because the mean of two effects is not correlated with, and thus independent of the difference between the same two effects. The direct test of the interaction in a 2x2 repeated-measures ANOVA with factors sequence (rr/cc) and pair type (ForwPair/OtherWithinPair) showed no interaction between pair type and sequence in amyg/hippo and pOFC (both $F(1,25)<0.5$, $p>0.5$), a trend-wise interaction in temporal pole ($F(1,25)=3.218$, $p=0.085$) and a significant interaction in mPFC ($F(1,25)=4.345$, $p=0.047$). In both of these latter cases, the interaction or trend-wise interaction arose because there was less, rather than more, suppression for forward pairs, and the winning model in a Bayesian rs-measures ANOVA contained only the factor sequence (tempPole: $BF_m=7.323$, $P(M|data) = 0.647$; mPFC: $BF_m=6.99$, $P(M|data) = 0.636$). Thus, in all cases, the rr-cc effect was not driven by only the subset of correctly ordered sequence pairs.

We note that this also implied that sequence fusion effects were not relying on transitions truly experienced during the pre-scan learning task, when only ForwPairs but no OtherWithinPairs were experienced. This points towards a more abstract representation of which elements belong or do not belong to the rewarded sequence.

Supplementary Note 8: Correct sequence order encoding depends on third- and fourth-order structure

We probed whether increases in BOLD activation in temporal pole and pOFC when transitioning through the correctly ordered rewarded sequence (i.e. A, AB, ABC, ABCD) were dependent on the third and fourth-order contingencies. Alternatively, this signal could be present even when only the pair structure (AB, BC, CD) is fulfilled. For this analysis, we focused on AB**C** versus xB**C** (a pair BC not preceded by A, bold indicates time-locking), and ABC**D** versus xxC**D** because A and AB by definition do not rely on higher-order chains. We extracted parameter estimates from a GLM that was almost identical to GLM1, except that instead of regressors (1) rr and (3) corrOrderRewSeq, we modelled one

regressor with all occurrences of xBC and xxCD, and one regressor with all occurrences of ABC/ABCD, plus other rr pairs of no interest here in their own separate regressor. We ran a 2x2 repeated-measures ANOVA on the resulting parameter estimates with factors Area (pOFC/tempPole) x HigherOrder (fulfilled yes/no, i.e. AB**C**/ABC**D** vs xB**C**/xxC**D**) and found a significant effect of HigherOrder ($F(1,25)=5.712$; $p=0.025$) but no effects of Area or interaction between Area x HigherOrder. Consistently, the winning model in a Bayesian rs-ANOVA was a model with HigherOrder factor but no other factors (BFm=4.936, P(M|data) = 0.552; **Fig 3c**).

Supplementary Note 9: Statistical learning of spatial distance and transition frequency is not driven by rewarded elements

To confirm that our measures of statistically acquired knowledge were indeed reflecting knowledge of relationships between *all* twelve stimuli, we repeated the analysis with a second GLM that split stimuli into those belonging to the rewarded and those belonging to the control sequence (RewSeq and ConSeq, respectively). The original GLM had one joint onset regressor for all twelve stimuli and parametric regressors across these twelve stimuli. In the control GLM, the onsets of rewarded and control stimuli were instead modelled separately and separately associated with parametric regressors for spatial distance and transition frequency. We note that this analysis would be expected to be slightly less powerful, as parametric regressors rely on 4 out of 12 stimuli and thus a third of the data in each case. Nevertheless, six out of eight effects reached significance (all $p<0.05$; **Suppl Fig 4d**) and the remaining two pointed in the same direction as in the initial analysis: for mPFC, transition frequency did not reach significance when fitted on RewSeq trials alone ($p=0.18$) and for amyg/hippo, transition frequency did not reach significance for ConSeq trials alone ($p=0.15$). Taken together, this is strong evidence that our measures of statistical learning were not driven by rewarded stimuli alone.

Supplementary Note 10: Transition frequency, not probability, is encoded in mPFC and amyg/hippo

Statistical associations (‘task model’) could be represented in terms of the conditional probabilities of transitions between stimuli given the initial stimulus or as pure state transition frequencies. The two types of representations have many similarities but the latter is arguably a more global, flexible, and abstract representation of the task space that is less dependent on the precise nature of the experiences during the time when it was acquired; the representation of transition from one stimulus to another is not normalized by the number of occasions the first stimulus has been experienced during learning. Intriguingly, activity in both mPFC and amyg/hippo was explained by a representation of state-transition frequencies (see above) but not by the conditional probability of a transition (all $p>0.05$), with a significant difference between these coding schemes in mPFC ($p=0.0039$,

t(25)=3.12), and a trend-wise difference in amyg/hippo (p=0.0575, t(25)=1.99; **Fig 4b**). Importantly, the evidence for a transition frequency representation remained significant when conditional transition probability was simultaneously included in the model. Note that the effects of conditional transition probability and pure transition frequency are dissociable from the expected stimulus frequency *per se*, which was only identified in visual areas (**Suppl Fig 4b**).

Supplementary Note 11: Dissociating tempPole-pOFC and hippo/amyg-mPFC networks

In the main text, we showed that BOLD responses in temporal pole and pOFC reflected the correctly ordered rewarded sequence, while mPFC and amyg/hippo carried knowledge of statistical relationships between all stimuli. To formally assess whether these two networks indeed carry different information, we ran additional analyses relating to the contrasts that differentiated between these areas: correct order and spatial/statistical transition. The first 2 x 2 ANOVA focused on the parameter estimates extracted from the correct order contrast in all four ROIs (**Fig 3b**). It included the factors Network (tempPole & pOFC versus amyg/hippo and mPFC) and Node (anterior/posterior, where pOFC and mPFC are the anterior nodes of the two networks both found within frontal cortex and tempPole and amgy/hippo are posterior nodes in the temporal lobe, respectively). There was a significant effect of Network (2 x 2 repeated measures ANOVA: F(1,25)=9.893, p=0.004) but no effect of Node or Network x Node (both p>0.2). Moreover, consistent with this result, a Bayesian repeated-measures ANOVA showed that the winning model indicated that only the factor Network significantly explained variation in activity between the regions (P(M|data)=0.65, BFm=7.34).

The second 2 x 2 x 2 ANOVA comprised the same conditions and the additional factor Contrast (spatial distance or transition frequency). This ANOVA focused on BOLD responses related to statistical knowledge (**Fig 4b**). Again, we found a significant effect of Network (F(1,25)=30.82, p<0.001) and no other significant main effects or interactions, only a trend for a Contrast x Network interaction (2 x 2 x 2 repeated-measures ANOVA: F(1,25)=3.472, p=0.074). The conclusions drawn from this analysis were bolstered by a Bayesian repeated-measures ANOVA that revealed three similarly good models of the data, all of which included a factor of Network (Model 1 had a main effect of Network only: P(M|data)=0.195, BFm=4.37; Model 2 included two main effects of Contrast and Network: P(M|data)=0.256, BFm=6.19; Model 3 contained three effects Contrast + Network + Network x Contrast: P(M|data)=0.251, BFm=6.02). In summary, in all cases, there was evidence for a difference between the activity patterns in the pOFC-tempPole and mPFC-amyg/hippo networks.

The identification of different response patterns in the two networks is consistent with a large body of work suggesting that there are major anatomical differences between pOFC and tempPole on the one hand and hippo/amyg and mPFC on the other hand. There are strong monosynaptic

connections within but not across these two networks[5–7]. For example, temporal pole and pOFC clusters, despite being in different lobes, are connected via the uncinate fascicle[8,9] while the hippocampus and mPFC are interconnected via the fornix[8,10]. These network connections are not just clear in tracer data but can also be appreciated using human resting-state data; there is strong within-network activity coupling (between pOFC and tempPole and between hippo/amyg and mPFC) but weaker across-network coupling (**Supplementary Figure 5**; source: Human Connectome Project (HCP) Data). Altogether, this provides robust evidence that the patterns of BOLD activation in the pOFC-temporal pole and amyg/hippo-mPFC were dissociable, with pOFC and temporal pole reflecting knowledge of the correctly ordered rewarded sequence and amyg/hippo and mPFC showing signatures of statistical learning. As previously noted, both types of learning, statistical and reward-learning, mediated aspects of stimulus-stimulus learning in our task. The dissociations between regions are less to do with the type of association but the mechanism of learning by which the association was derived.

Supplementary Note 12: No evidence for behavioral effects of spatial spreading of reward

We examined whether the spatial spread of reward effect identified in the amygdala might be reflected in the behaviour of the participants. We first examined the choice data collected after each block of the pre-scan learning task and checked if we had trials where participants made choices between a neighbour of D and a non-RS element. We had an average of eleven such trials per choice block (in a total of 12 blocks per day) but choices did not differ significantly from chance ($p>0.4$ on both Day1 and Day2 of training), not even when looking at only the very first block of training. Similarly, there was no RT speeding of responses to neighbours of D compared to neighbours of D' during the task performed in the scanner (paired t-test; $p=0.14$).

Supplementary Table 1, related to Figure 2

Complete table of BOLD activations that survived cluster-correction for rr-cc (Figure 2a)

**Region**	**MNI coordinate (x,y,z)**	**Peak activation**	
Temporal pole	48,10,-28	Z=4.19	Shown in Fig 2a
Cerebellum	-28,-84,-34	Z=4.02	
mPFC	0,40,-6	Z=4.0	Shown in Fig 2a
Posterior OFC	30,18,-22	Z=3.73	Shown in Fig 2a
Occipital pole	-6,-91,23	Z=3.43	

Supplementary Table 2, related to Figures 2-4

Table summarizing all effects related to pairs of stimuli

**Pairs of stimuli included in analysis**	**Contrast**	**Sign of effect**	**Regions in which this effect is observed**	**Figures**
AB, BC, CD, BA, CB, DC, AC, AD, BD, CA, DA, CA (and equivalent ones for the ConSeq)	RR-CC	Smaller BOLD for repetitions within the RewSeq compared to the ConSeq (consistent with greater repetition suppression within RewSeq)	tempPole<br>pOFC<br>mPFC<br>amyg/hippo	Figure 2/Supplementary Figure 2
AB, ABC and ABCD<br>vs<br>A'B', A'B'C' and A'B'C'D'	Correct Order RewSeq-ConSeq	Stronger BOLD for RewSeq compared to ConSeq consistent with build-up of reward expectation/reward proximity	tempPole<br>pOFC	Figure 3/ Supplementary Figure 3
Pairs with close vs far spatial proximity<br><br>Pairs with frequent vs infrequent transition probability	spatDist (parametric)<br><br>transFreq (parametric)	Stronger BOLD for spatially close or likely transitions (not consistent with repetition suppression)	mPFC<br>amyg/hippo	Figure 4/ Supplementary Figure 4

Supplementary figures

Supplementary Figure 1

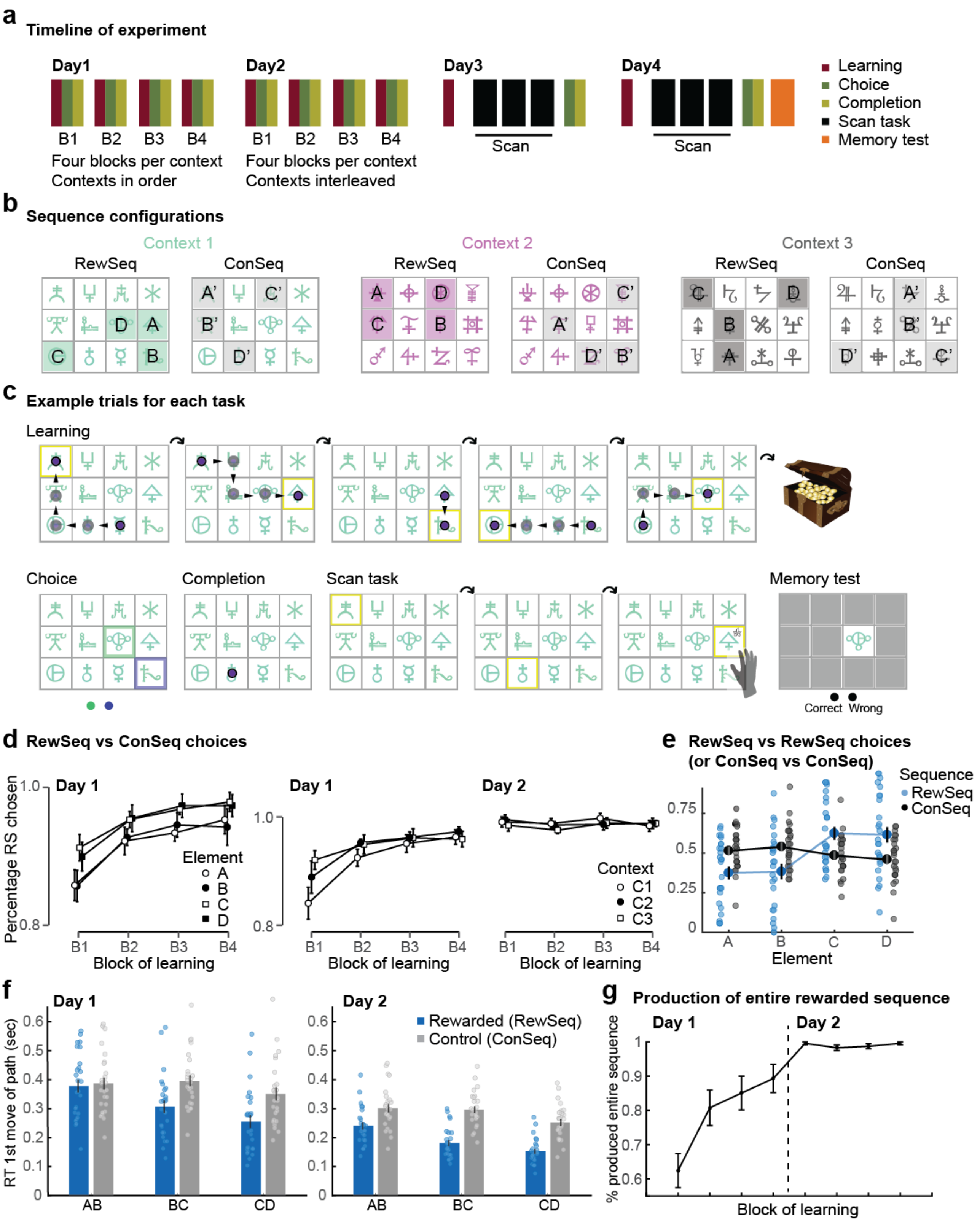

Supplementary Figure 1, related to Figure 1: further task details and behavioral results: preference for rewarded sequence elements

a, Timeline of the experiment: four sessions were completed within a week. The first two were behavioral and involved learning the reinforced sequence. The third and fourth sessions involved a different task performed during fMRI scanning. **b**, RewSeq and ConSeq combinations used in the three contexts; in half the participants ConSeq and RewSeq were swapped. **c**, Exemplary screen displays for each part of the experiment. During learning, subjects moved a dot (agent) around a 3x4 grid towards highlighted stimuli. A reward occurred once they had visited a specific sequence of stimuli (ABCD; RewSeq). During the choice task, two stimuli were highlighted and subjects indicated which one they associated more with reward. During the completion task, subjects moved a randomly placed dot around but no stimuli were highlighted. They had to complete their hypothesized rewarded sequence by pressing a button while occupying appropriate stimuli. During the scanning task, a series of highlighted stimuli were shown. Subjects had to press a button when a flower appeared on the highlighted stimulus. An unannounced memory task probed participants' recall at the end of testing. Stimuli could be shown in the correct color/position or either wrong color or wrong position. **d**, Choices between any RewSeq and any equally frequent ConSeq stimulus. (Left) RewSeq elements were preferred over ConSeq elements (50%=chance) and this manifested earlier for C/D compared to A/B stimuli. (Right) For the four blocks on day 1+2, the percentage any RewSeq stimulus was chosen over any ConSeq element are shown for the three contexts. Accuracy in contexts 2 and 3 started at higher levels than block 1 (task set). **e**, Choices between elements of the same sequence (RewSeq vs RewSeq or ConSeq vs ConSeq): later elements (C,D over A,B) were preferred for RewSeq but not for ConSeq. This is consistent with RL occurring backwards from reward. **f**, The anticipation of correct sequence successors improved from day 1 to day 2 (compare Figure 1b). **g**, Percentage of times the entire RewSeq was produced during the completion task. Error bars denote SE.

Supplementary Figure 2

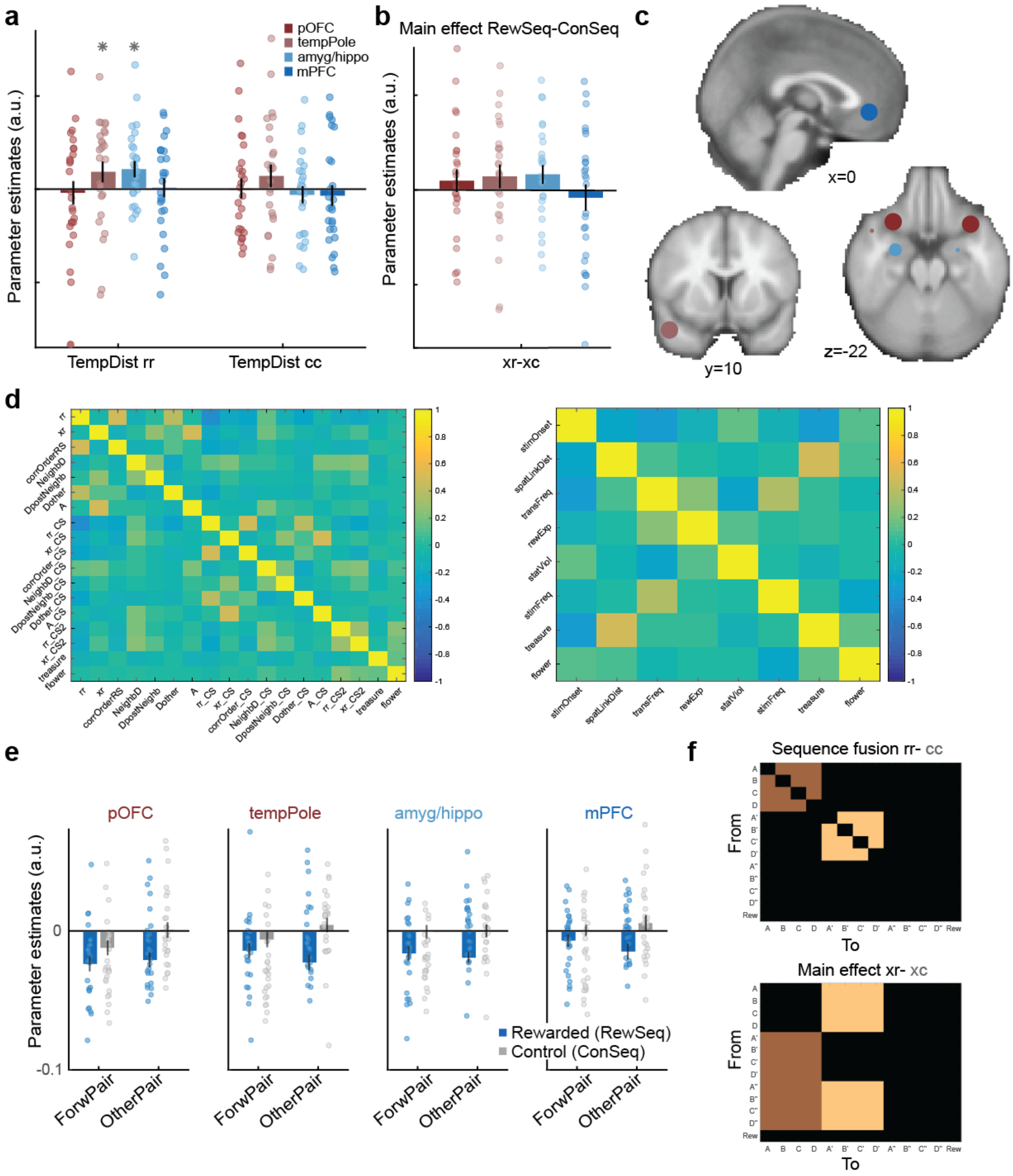

Supplementary Figure 2, related to Figure 2: temporal modulation of effects, ROI definition and GLM orthogonality

a, The modulation of BOLD repetition suppression as a function of the temporal gap between two successive RewSeq or ConSeq elements was only present in temporal pole and amyg/hippo, and only for the second of two RewSeq elements (rr) but not the second of two ConSeq elements (cc); left is as

shown in **Fig 2**, right is added for completeness; * indicates $p<0.05$ in one-sample t-test. **b**, The main difference in BOLD response to rewarded and control elements was not significant in any of our ROIs (xr-xc), suggesting that the ROI-defining contrast (rr-cc) indeed captured cross-stimulus repetition suppression effects, and thus probed relationships within the rewarded and control sequences, rather than main activation differences. Main activation differences are tested here based on trials where a rewarded stimulus was preceded by any non-rewarded stimulus (xr; and the same for the control sequence: xc). This test was independent of the ROI-defining contrast. **c,** ROI spheres centered on the peak locations of the activations shown in **Fig 2** in temporal pole (light red), posterior OFC (dark red), amygdala-hippocampus border (light blue) and mPFC (dark blue); these ROIs are used for the remainder of the manuscript. **d**, Correlation (Pearson's r) between regressors of the two main GLMs (see Methods). GLM1 (left) was used for the analyses shown in Figs 2 and 3, 5 and 6, and GLM2 (right) was used for the analyses shown in Figs 4 and 6. **e**, Splitting pairs of the rewarded and control sequence (i.e. rr and cc) into the corresponding 'forward pairs' (AB, xBC, xxCD) and 'other within-sequence pairs' (BA, CB, DC, AD, DA, AC, CA, BD, DB) showed that both subsets contributed to the cross-stimulus suppression effects in all ROIs. Thus, sequence fusion effects were not driven by only the subset of correctly ordered (forward) pairs. **f**, The sequence fusion and main effect contrasts are illustrated in the full space of all transitions (row = from, column = to). Compare with **Supplementary Figure 4c** for another contrast related to transitions. Error bars denote SE.

Supplementary Figure 3

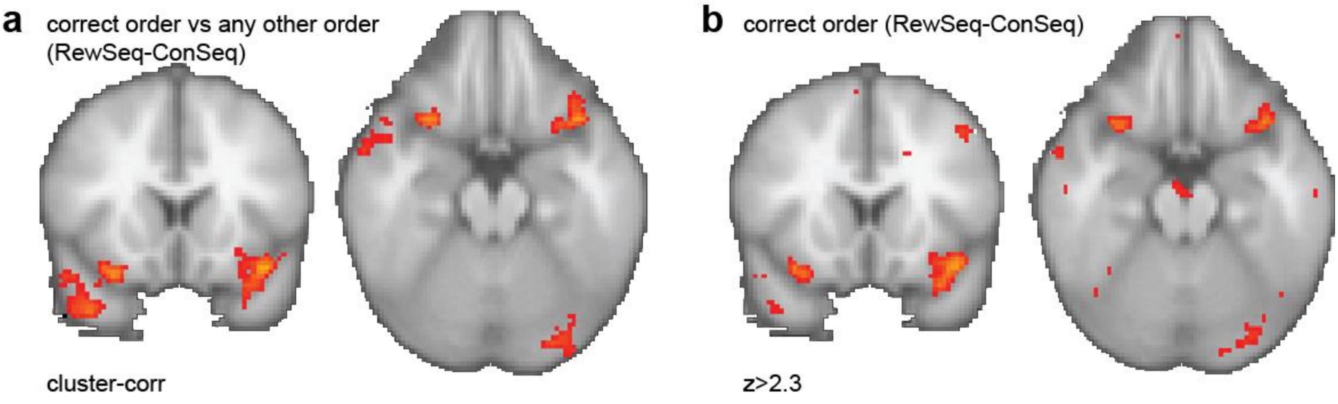

Supplementary Figure 3, related to Figure 3: whole-brain results for correct sequence order

Whole-brain results for the encoding of correct sequence order. **a**, RewSeq elements that follow the correct order are contrasted with RewSeq elements that came in any other order and the same comparison for the ConSeq is subtracted. This highlights strong bilateral activation in pOFC and unilateral activation in temporal pole (all cluster-corrected). **b**, RewSeq elements that follow the correct order are contrasted with ConSeq elements that follow the correct order directly. This contrast is shown at a lower threshold (z>2.3) but reveals a similar pattern of results.

Supplementary Figure 4

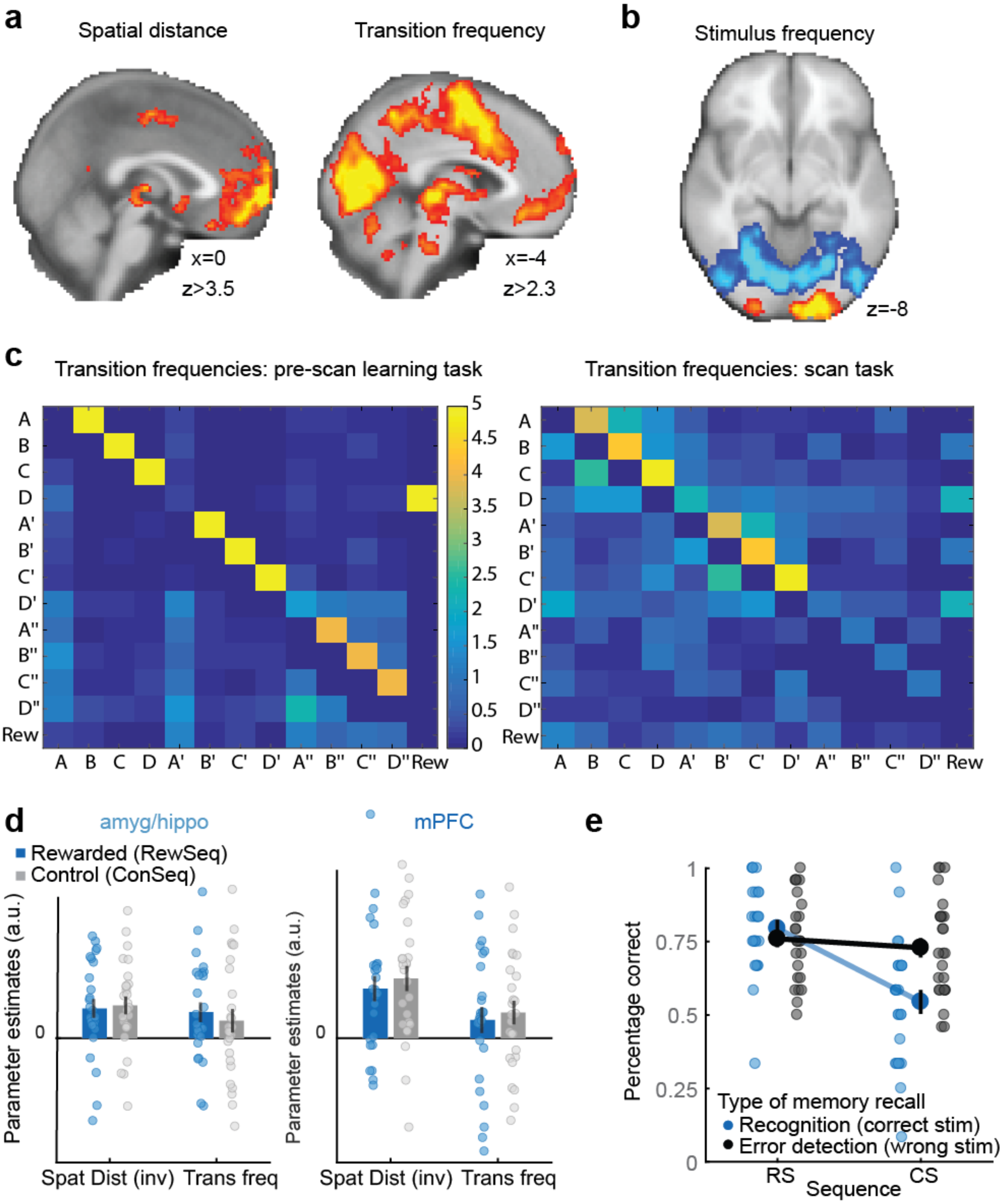

Supplementary Figure 4, related to Figure 4: whole-brain results for spatial distance and transition frequency

a, Whole-brain results for spatial distance and transition frequency. **b**, Whole-brain contrast for stimulus frequency. **c**, Transition frequencies experienced in the pre-scan learning task (left) and during scanning (right). **d**, Splitting up spatial distance and transition frequency contrasts for stimuli of the rewarded versus control sequence showed no significant differences between RewSeq and ConSeq. Thus, spatial distance and transition frequency effects were indeed present across *all* stimuli. **e**, Performance on post-scan memory test where stimuli were either shown in the correct position and color, probing recognition memory, or in the wrong color or position, probing error detection of the 3x4 layout. Participants' judgement on whether stimuli were correct or wrong was more accurate for stimuli shown in the wrong color or position and similar for stimuli of either sequence. When asked to judge correct stimuli, however, performance was significantly higher for reinforced elements of RewSeq. Error bars denote SE.

Supplementary Figure 5

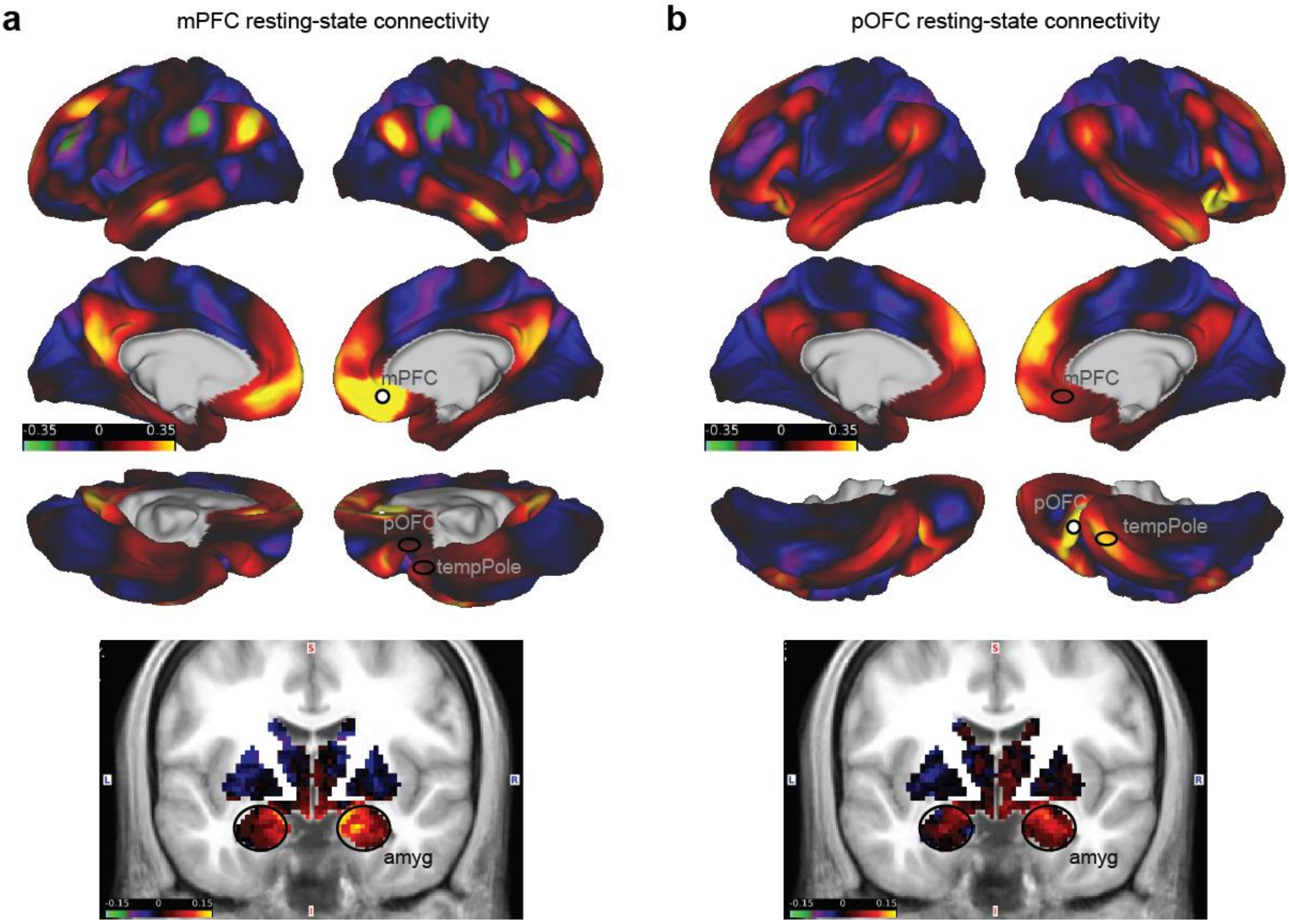

Supplementary Figure 5, related to Figure 3 and 4: strong within-network activity coupling (between pOFC and tempPole and between hippo/amyg and mPFC)

Whole brain resting-state functional coupling based on the average of 200 people from the Human Connectome Project (HCP) highlights coupling within each network (temporal pole and pOFC; mPFC and amyg/hippo) but less so across networks at rest. (a) For example, looking at resting state connectivity of mPFC (white dot labelled mPFC), it is clear that activity is strongly coupled with that in amyg/hippo, PCC and some other regions, but there is weak or no coupling to temporal pole and pOFC (hotter colors indicate increased resting-state coupling; locations of ROIs are highlighted with circles). (b) By contrast, resting-state connectivity of pOFC (white dot) shows some mPFC and amyg/hippo coupling but particularly strong connectivity with temporal pole.

Supplementary Figure 6

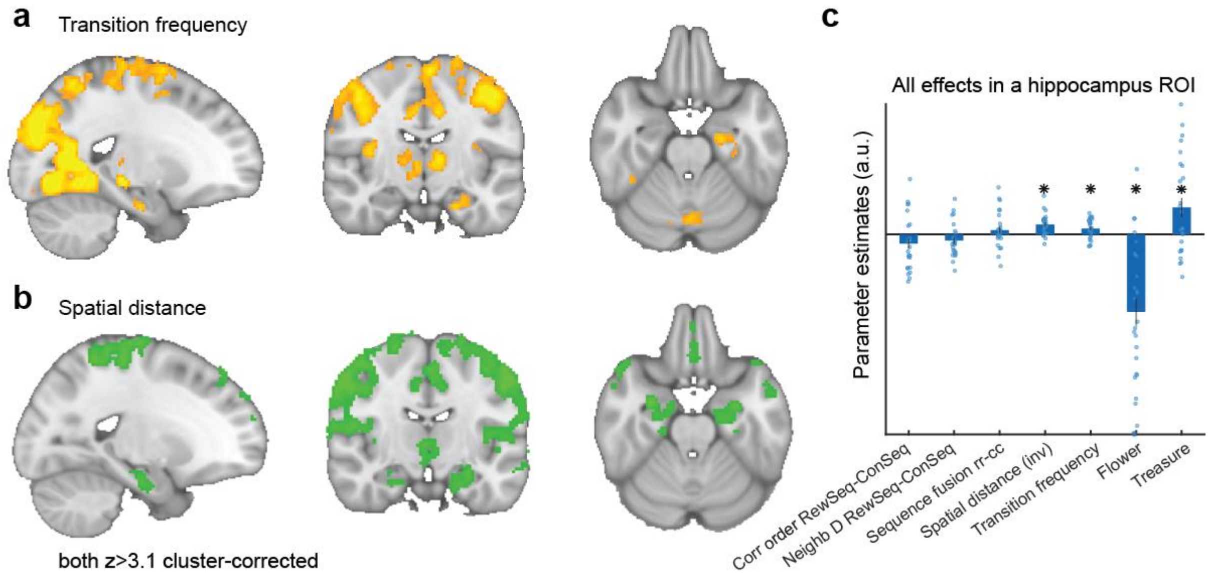

Supplementary Figure 6, related to Figure 3, 4 and 6: BOLD signatures of statistical learning in the hippocampus

The hippocampus showed effects of transition frequency (**a**) and spatial distance (**b**); contrasts are the same as in **Supplementary Figure 4a** but with views showing the hippocampus (both z>3.1/p<0.001 cluster-corrected). **c**, All effects in the hippocampus are illustrated for a spherical ROI centered on a coordinate taken from Garvert et al., eLife, 2017. This shows BOLD signatures related to statistical learning but no knowledge of the rewarded over and above the control sequence; error bars denote SE; * indicates p<0.05 in one-sample t-test.

Supplementary References

1. Turk-Browne, N. B., Jungé, J. & Scholl, B. J. The automaticity of visual statistical learning. *J. Exp. Psychol. Gen.* **134**, 552–564 (2005).
2. Batterink, L. J. & Paller, K. A. Online neural monitoring of statistical learning. *Cortex* **90**, 31–45 (2017).
3. Siegelman, N., Bogaerts, L., Kronenfeld, O. & Frost, R. Redefining 'Learning' in Statistical Learning: What Does an Online Measure Reveal About the Assimilation of Visual Regularities? *Cogn. Sci.* **42 Suppl 3**, 692–727 (2018).
4. Kim, R., Seitz, A., Feenstra, H. & Shams, L. Testing assumptions of statistical learning: is it long-term and implicit? *Neurosci. Lett.* **461**, 145–149 (2009).
5. Kondo, H., Saleem, K. S. & Price, J. L. Differential connections of the perirhinal and parahippocampal cortex with the orbital and medial prefrontal networks in macaque monkeys. *J. Comp. Neurol.* **493**, 479–509 (2005).
6. Carmichael, S. T. & Price, J. L. Limbic connections of the orbital and medial prefrontal cortex in macaque monkeys. *J. Comp. Neurol.* **363**, 615–641 (1995).
7. Saleem, K. S., Kondo, H. & Price, J. L. Complementary circuits connecting the orbital and medial prefrontal networks with the temporal, insular, and opercular cortex in the macaque monkey. *J. Comp. Neurol.* **506**, 659–693 (2008).
8. Croxson, P. L. *et al.* Quantitative investigation of connections of the prefrontal cortex in the human and macaque using probabilistic diffusion tractography. *J. Neurosci. Off. J. Soc. Neurosci.* **25**, 8854–8866 (2005).
9. Schmahmann, J. D. & Pandya, D. N. The complex history of the fronto-occipital fasciculus. *J. Hist. Neurosci.* **16**, 362–377 (2007).
10. Aggleton, J. P., Wright, N. F., Rosene, D. L. & Saunders, R. C. Complementary Patterns of Direct Amygdala and Hippocampal Projections to the Macaque Prefrontal Cortex. *Cereb. Cortex N. Y. N 1991* **25**, 4351–4373 (2015).
