# Supplementary material for: Multiple associative structures created by reinforcement and incidental statistical learning mechanisms
Source: Nat Commun. 2019 Oct 23;10:4835. doi: 10.1038/s41467-019-12557-z (PMC6811627; doi:10.1038/s41467-019-12557-z)
Supplement: Supplementary file 3 — Reporting Summary [file 41467_2019_12557_MOESM3_ESM.pdf]

nature research

Corresponding author(s): Miriam Klein-Flugge

Reporting Summary

Nature Research wishes to improve the reproducibility of the work that we publish. This form provides structure for consistency and transparency in reporting. For further information on Nature Research policies, see Authors & Referees and the Editorial Policy Checklist.

Statistical parameters

When statistical analyses are reported, confirm that the following items are present in the relevant location (e.g. figure legend, table legend, main text, or Methods section).

n/a	Confirmed	
☐	☒	The exact sample size (n) for each experimental group/condition, given as a discrete number and unit of measurement
☐	☒	An indication of whether measurements were taken from distinct samples or whether the same sample was measured repeatedly
☐	☒	The statistical test(s) used AND whether they are one- or two-sided<br>*Only common tests should be described solely by name; describe more complex techniques in the Methods section.*
☐	☒	A description of all covariates tested
☐	☒	A description of any assumptions or corrections, such as tests of normality and adjustment for multiple comparisons
☐	☒	A full description of the statistics including central tendency (e.g. means) or other basic estimates (e.g. regression coefficient) AND variation (e.g. standard deviation) or associated estimates of uncertainty (e.g. confidence intervals)
☐	☒	For null hypothesis testing, the test statistic (e.g. F, t, r) with confidence intervals, effect sizes, degrees of freedom and P value noted<br>*Give P values as exact values whenever suitable.*
☐	☒	For Bayesian analysis, information on the choice of priors and Markov chain Monte Carlo settings
☒	☐	For hierarchical and complex designs, identification of the appropriate level for tests and full reporting of outcomes
☐	☒	Estimates of effect sizes (e.g. Cohen's d, Pearson's r), indicating how they were calculated
☐	☒	Clearly defined error bars<br>*State explicitly what error bars represent (e.g. SD, SE, CI)*

Our web collection on statistics for biologists may be useful.

Software and code

Policy information about availability of computer code

Data collection	The psychophysics toolbox (Brainard, 1997) (within Matlab) was used to collect the data.
Data analysis	Data analysis was conducted in Matlab 2014b/2018a and JASP (https://jasp-stats.org).

For manuscripts utilizing custom algorithms or software that are central to the research but not yet described in published literature, software must be made available to editors/reviewers upon request. We strongly encourage code deposition in a community repository (e.g. GitHub). See the Nature Research guidelines for submitting code & software for further information.

Data

Policy information about availability of data

All manuscripts must include a data availability statement. This statement should provide the following information, where applicable:

- Accession codes, unique identifiers, or web links for publicly available datasets
- A list of figures that have associated raw data
- A description of any restrictions on data availability

The data underlying all figures are provided as part of the Open Science Framework (OSF; https://osf.io/y325a; Digital Object Identifier (DOI): http://doi.org/10.17605/OSF.IO/Y325A). The OSF project contains a zip folder with all source Data files (.mat), a spreadsheet detailing the names of the source data files and all scripts relevant for producing the figure (make_figX.m). Unthresholded fMRI maps of all contrasts are available on Neurovault (https://identifiers.org/

neurovault.collection:5765).

Field-specific reporting

Please select the best fit for your research. If you are not sure, read the appropriate sections before making your selection.

☐ Life sciences ☒ Behavioural & social sciences ☐ Ecological, evolutionary & environmental sciences

For a reference copy of the document with all sections, see nature.com/authors/policies/ReportingSummary-flat.pdf

Behavioural & social sciences study design

All studies must disclose on these points even when the disclosure is negative.

Study description	Data are quantitative experimental data.
Research sample	Twenty-seven participants (11 female, age 19-35, mean 26, SE 0.77) including students, staff and public members from around Oxford took part in the study.
Sampling strategy	Random sampling was used. Our sample size was based on similar experimental paradigms and the effect sizes typically reported for this kind of study, indicating sufficient power.
Data collection	Participants performed some aspect of the task in front of a computer monitor with the researcher present in the same room (behavioural sessions 1 and 2), and other parts of the task inside an MRI scanner in a room adjacent to the researcher and radiographers (MRI sessions 3 + 4).
Timing	Data collection took place between July 2015 and March 2017.
Data exclusions	One participant failed to complete the experiment because he fell asleep repeatedly in the scanner, and was thus excluded from the data analysis.
Non-participation	No participants dropped out or declined participation.
Randomization	All participants were collapsed into one experimental group and performed the same task, but for the purpose of counterbalancing the precise way the stimuli were presented across participants, participants were randomly assigned to one of two groups.

Reporting for specific materials, systems and methods

Materials & experimental systems

n/a	Involved in the study	
☒	☐	Unique biological materials
☒	☐	Antibodies
☒	☐	Eukaryotic cell lines
☒	☐	Palaeontology
☒	☐	Animals and other organisms
☐	☒	Human research participants

Methods

n/a	Involved in the study	
☒	☐	ChIP-seq
☒	☐	Flow cytometry
☐	☒	MRI-based neuroimaging

Human research participants

Policy information about studies involving human research participants

Population characteristics	Twenty-seven participants (11 female, age 19-35, mean 26, SE 0.77): screening criteria included no history of neurological or psychiatric disorder, normal or corrected-to-normal vision and suitability for undergoing MRI scanning.
Recruitment	Participants were recruited through email advertisements, poster boards, and the departmental database.

Magnetic resonance imaging

Experimental design

Design type	Event-related design
Design specifications	Learning task/choice task/completion task: Two behavioural days; 12 blocks per day, 96 stimuli/54 choices/3 completion trials per block; two scan days: 3 blocks per day (same trial numbers); MRI task: two days, three blocks per day, 414 stimuli per block. Post-scan memory task (1 block): 108 choices. Timings were self-paced in the behavioural task and ITIs were drawn from truncated Poisson-distribution: mean 2.8s, range 1.5-10s for the scan task. See Figure 1 and S1 for full experimental details.
Behavioral performance measures	All button presses and response times were recorded. We performed a number of analyses to show participants performed the task correctly (e.g. Fig 1, S1, number of flowers caught; post-scan memory test). For details see manuscript. Repeated-measures ANOVAs (frequentist and Bayesian) were used to determine significant main effects and interactions.

Acquisition

Imaging type(s)	Functional and structural
Field strength	3 Tesla
Sequence & imaging parameters	A Siemens Prisma 3T MRI scanner was used to acquire multiband T2*-weighted echo planar imaging (EPI) volumes with blood oxygenation-level–dependent (BOLD) contrast. Slices were acquired in interleaved order and at an oblique angle of -30˚ to the AC-PC line to reduce signal dropout in orbitofrontal cortex. The voxel size was 2x2x2mm with a 1 mm gap; TE=32.4 ms; repetition time=1354ms; flip angle=74°; number of slices: 72. One run (~20 minutes) contained approximately 850 volumes. A fieldmap (2x2x2mm) was obtained for each subject to allow for corrections in geometric distortions. In one of the two scanning sessions of each participant, a structural MPRAGE scan was acquired with 192 slices; slice thickness=1 mm; TR=1900 ms; TE=3.94 ms; voxel size=1x1x1 mm.
Area of acquisition	Whole brain scan

Diffusion MRI ☐ Used ☒ Not used

Preprocessing

Preprocessing software	Image preprocessing was implemented in FMRIB Software Library (FSL) (Smith et al., 2004) and consisted of bias correction using the bias field obtained from segmentation (Zhang et al., 2001), motion correction (Jenkinson, 2002), distortion correction using fieldmaps, brain extraction, high-pass filtering, and spatial smoothing with a 5 mm FWHM kernel. A hard regression was performed to regress out noise explained by 24 motion regressors (the original six produced by FLIRT, their derivatives, and the resulting 12 regressors squared; see e.g., Glasser et al., 2013) and by 33 physiological noise regressors created using the PNM toolbox (because of the short TR, these were not voxel-wise regressors: oc=4; or=4; multc=2; multr=2; rvt; (Brooks et al., 2008)). In addition, conservative independent component analysis was used to identify and remove obvious artifacts (using MELODIC in Fmrib's Software Library; http://fsl.fmrib.ox.ac.uk/) and ICA noise components were regressed out of the data using a soft regression.
Normalization	Images were registered to the high-resolution structural image (BBR) and then the standard MNI152 template using nonlinear registration (12 degrees of freedom) (Jenkinson and Smith, 2001).
Normalization template	The MNI152 template was used.
Noise and artifact removal	A hard regression was performed to regress out noise explained by 24 motion regressors (the original six produced by FLIRT, their derivatives, and the resulting 12 regressors squared; see e.g., Glasser et al., 2013) and by 33 physiological noise regressors created using the PNM toolbox (because of the short TR, these were not voxel-wise regressors: oc=4; or=4; multc=2; multr=2; rvt; (Brooks et al., 2008)). In addition, conservative independent component analysis was used to identify and remove obvious artifacts (using MELODIC in Fmrib's Software Library; http://fsl.fmrib.ox.ac.uk/) and ICA noise components were regressed out of the data using a soft regression.
Volume censoring	No volume censoring was used.

Statistical modeling & inference

Model type and settings	We used univariate models. Contrast were estimated at the first level for each run of each day in each subject. At the second level, the sessions from the same day in each subject were combined using fixed-effects (second stage); at the third level, the sessions from the two days of each subject were combined using FLAME1 (mixed-effects (Beckmann et al., 2003)). Finally, the fourth level corresponded to the group analysis and used FLAME1 to combine across subjects.
Effect(s) tested	The contrasts of interest in GLM1 (see Methods) were rr-cc (Fig 2a), which probes any differences in BOLD to the second of two successive stimuli from RS compared to CS (for more details, see Results); corrOrderRS-corrOrderCS which probes differences between RS sand CS when transitioning through the correctly ordered associations (Fig 3b and S3). One other contrast of interest in GLM1 related to the spreading of reward in space: we compared spatial neighbors of D with spatial neighbors of D' (Fig 5). And a final contrast compared the encoding of the starting elements A and

A' (Fig 6). The relevant contrasts in GLM2 (see Methods) were the parametric effect of spatial link distance and transition frequency (Fig 4) which were taken as measures for statistical learning.

Specify type of analysis: ☐ Whole brain ☐ ROI-based ☒ Both

Anatomical location(s): Anatomical ROIs for ventral striatum (nucleus accumbens) and amygdala were constructed from the Harvard Subcortical Atlas (probability threshold 0.25) because of a priori hypotheses about these regions.

Statistic type for inference (See Eklund et al. 2016): Cluster-based family-wise error correction was used. We originally performed analyses with a cluster-forming threshold of p<0.1 (z>2.3) but repeated them with a more conservative cluster-forming threshold of p<0.001 (z>3.1), following recent recommendations (Eklund et al., 2016). This did not change the key results (see Methods).

Correction: FWE was used (p<0.05).

Models & analysis

n/a	Involved in the study
☒	☐ Functional and/or effective connectivity
☒	☐ Graph analysis
☒	☐ Multivariate modeling or predictive analysis
